# Association of Household Environment and Prevalence of Anemia Among Children Under-5 in India

**DOI:** 10.3389/fpubh.2014.00196

**Published:** 2014-10-20

**Authors:** Annu Baranwal, Anshu Baranwal, Nobhojit Roy

**Affiliations:** ^1^Environmental Health Resource Hub, School of Habitat Studies, Tata Institute of Social Sciences, Mumbai, India; ^2^International Institute for Population Sciences, Mumbai, India; ^3^Department of Public Health Sciences, Karolinska Institutet, Stockholm, Sweden

**Keywords:** children, anemia, environment, health, India

## Abstract

**Objective:** The study explores the association between the household environment and the prevalence of anemia among children under the age of 5 years in India.

**Data and methodology:** The study is based on 52,868 children under the age of 5 years, included in India’s National Family Health Survey-3. The outcome variable was the prevalence of anemia. To understand the role of environment in determining child anemia, step wise logistic regression models consisting of environmental, child, socio-economic, and media exposure variables were applied.

**Results:** The occurrence of childhood anemia was higher in the North Eastern and Eastern regions compared to all other regions of India. Unclean fuel use, poor toilet facilities, staying in non-concrete house, exposure to smoking were important variables determining the prevalence of anemia. Smoking, when it was controlled with only socio economic factors, showed lesser impact on anemia, but when it got adjusted with socio-economic, child, and media variables together it showed an important impact as it increased the risk of anemia.

**Conclusion:** Children under 5 years of age generally stay inside their house and are more exposed to the household environment. Thus, among these children there are multiple risk factors causing anemia along with the nutritional deficiencies. Better resources are needed to educate the public and to increase awareness for improved hygiene, sanitation and housing facilities, health and nutrition, etc. Along with a wider program to manage nutritional deficiency, anemia in children <5 years, there should be a holistic approach toward anemia control inculcating household environmental conditions and socio economic determinants.

## Introduction

Anemia is the major health problem in India especially among children and women. The National Family Health Survey (NFHS)-3 in India shows that around 70% of children are anemic in <5 years of age group and has increased around 5% point from 1999 to 2006. Although there is high impact of background characteristics on severity of anemia, it is widespread in every group and every state. More than half of the children are anemic even if their mother has 12 or more years of education or is in highest wealth quintiles (1). Anemia is considered to be an important contributing factor to the global burden of disease. Affecting both developed and developing countries, it has an impact on not only human health and productivity but in the process it affects the socio-economic development of a nation. Though preventable, anemia still remains widely prevalent in the world. The WHO Global Database on Anemia estimated the prevalence to be 25% of the general population. It is known to affect all age groups, but pregnant women and pre-school children are at greater risk. It affects 1.62 billion people globally with about 293 million children under-5 years (i.e., 47% of the affected population) and of this children population, more than 65% is in South-East Asia (this is second only to Africa) (2).

Anemia in children is influenced by structural and environmental factors, community, household factors, and individual’s health and nutritional level. Anemia in pre-school children has a negative effect on cognition, motor development and growth, academic performance, immunity, and susceptibility to infections (3). These threats to health in earlier life are determinants of other health problems in later life (4).

Most of the previous studies done on anemia among children are based on socio economic and nutritional correlates. Very few studies have seen the combined effect of socio economic and environmental factors on anemia among children. This study thus examines the association of the household environment with the prevalence of anemia among children <5 years of age, in India.

## Data Source

**Figure 1:**
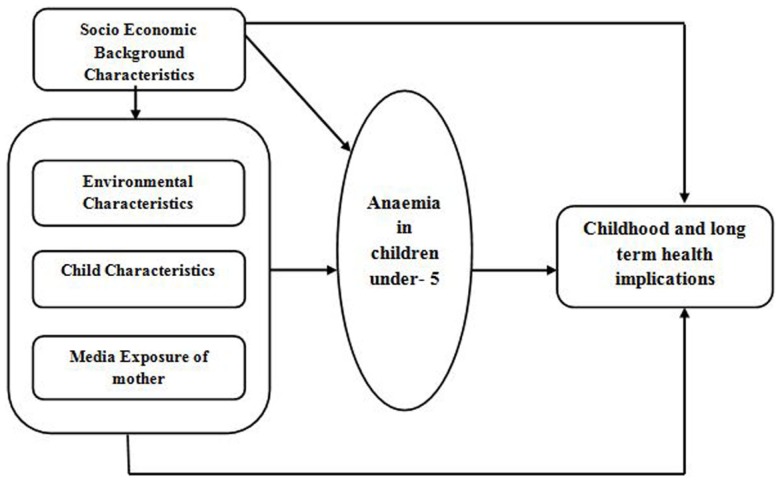
**Conceptual framework for the study**.

The data set used for this study was the NFHS-3 (2005–06). The NFHS is a large scale, multi-round survey conducted in a representative sample of households throughout India. Three rounds have been conducted since the first survey in 1992–1993. The survey provided state and national information for India on fertility, infant and child mortality, the practice of family planning, maternal and child health, reproductive health, nutrition, anemia, utilization, and quality of health and family planning services. Each successive round of the NFHS has had two specific goals: (a) to provide essential data on health and family welfare needed by the Ministry of Health and Family Welfare (MOHFW) and other agencies for policy and program purposes, and (b) to provide information on important emerging health and family welfare issues. The MOHFW, Government of India, designated International Institute for Population Sciences (IIPS), Mumbai, as the nodal agency, responsible for providing coordination and technical guidance for the survey. IIPS collaborated with a number of Field Organizations (FO) for survey implementation. Each FO was responsible for conducting survey activities in one or more states covered by the NFHS. Technical assistance for the NFHS was provided mainly by ORC Macro (USA) and other organizations on specific issues. The funding for different rounds of NFHS was provided by USAID, DFID, the Bill and Melinda Gates Foundation, UNICEF, UNFPA, and MOHFW, GOI. Information collected covered 52,868 children under the age of 5 years from a sample of 124,385 women between the ages of 15 and 49 years in all 29 states. (Children from ever married women included in the study.) Since anemia is a serious health problem in India, NFHS-3 undertook direct measurement of the hemoglobin levels of all children under age 5 years, women age 15–49 years, and men age 15–54 years. The prevalence of anemia among children was estimated by measuring the hemoglobin using the Hemo Cue^TM^ Hb 201+ analyzer. This point-of-care quantitative hemoglobin testing system uses a single drop of blood from a finger prick (or heel prick in the case of infants under 6 months old), drawn into a curette, and then inserted into a portable, battery-operated instrument. To understand the household environment, a number of questions from the household questionnaire were asked to the head of the household. Figure 1 shows the conceptual framework for the study.

## Variables and Methodology

### Exposure variables

In this study, prevalence of anemia was calculated by four sets of variables, namely environmental variables, child related variables, socio-economic variables, and media exposure of women.

Environmental variables had seven main components, which were: source of drinking water (improved source of drinking water- piped water, piped into dwelling, piped to yard plot, public tap/stand pipe, tube well water, tube well or borehole, protected well, protected spring, rain water, bottled water; not improved source of drinking water-unprotected well, surface water, river/dam/lake/ponds/stream/canal/irrigation channel, tanker truck, cart with small truck), toilet facility (toilet facility available and toilet facility not available), type of house (Kaccha house – made from mud, thatch, or low quality materials, Semi pucca house – made from partly low quality and partly high quality material, Pucca house – made entirely from high quality materials including the floor, roof, and exterior walls), type of cooking fuel (clean cooking fuel – electricity, LPG/natural gas/biogas, kerosene; unclean cooking fuel – coal/ignite/charcoal, wood, straw/shrubs/grass, agricultural crop, animal dung), household structure (nuclear and non-nuclear), environmental tobacco smoke (ETS) (whether anyone in the house smoked – yes or no), and seasonality (winter, summer, and rains).

Child related variables included – sex of child (boy or girl), age of child (<6 months, 6 = 23 months, 24–59 months), birth order of child (1–4), stunting (normal or stunted), duration of breastfeeding (never been breastfed, <6 months, 6 months and above, still being breastfed), treatment for parasitic infection (yes or no).

Socio-economic variables were – place of residence (urban, rural), caste (others, general, SC/ST, OBC), religion (hindu, muslim, others), mother’s education (no education, primary, secondary, and above), mother’s working status (not working, working), wealth index (rich, middle class, poor), geographical region: north (mainly the mountainous states along with states of north-west India that included plain and dry land), central (plain and plateau terrain), east, northeast (plain land and rugged terrain with hills and mountains), west (coastal plain, and plateau), and south (coastal plain and plateau).

Impact of media exposure on women was determined by whether they read newspapers/magazines or whether they watched television or heard radio. It was entered as “yes” or “no.”

### Outcome variable

Anemia was a dichotomous dependent variable where 1 was coded for those children who had anemia and 0 was otherwise. So the odds ratio explained the chance or probability of suffering from anemia according to the predictor variables.

To understand the role of environment in anemia in children, step wise logistic regression models consisting of environmental, child, socio economic, and media exposure variables were applied at all India level, making four consecutive models on SPSS package (version 20.0) (SPSS, Inc., Chicago IL, USA).

## Results

### Bivariate analysis

**Figure 2:**
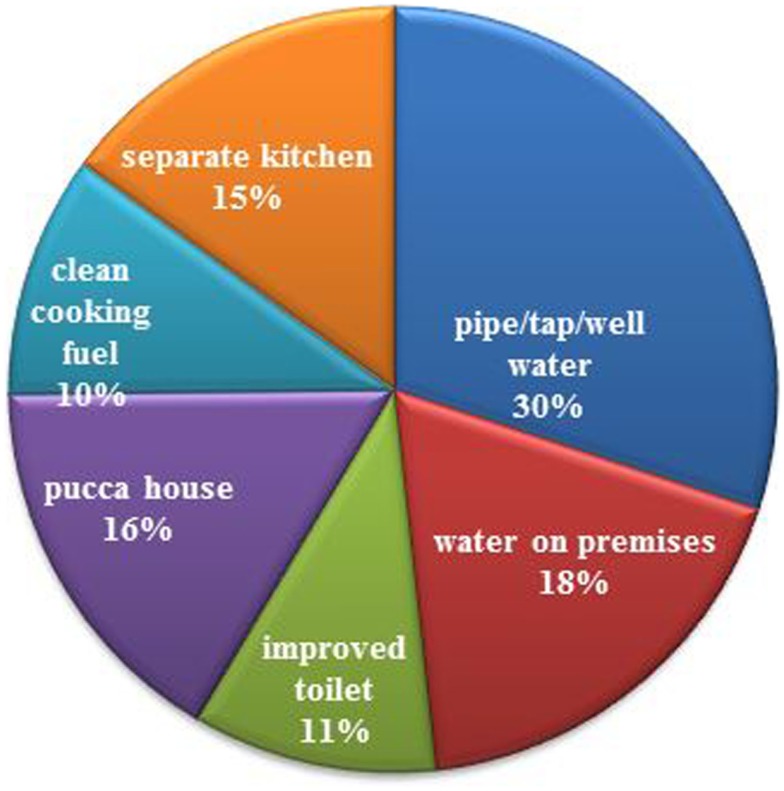
**Percentage of households by built environment**.

Building environment: from Figure 2, it is inferred that none of the positive environmental factors were well beyond 30% of the provision of drinking water by pipe/tap/well; these figures were substantially lower in rural settings. Just about 30% of the population had improved toilet facilities (not shared flush and pit toilet) and used clean fuel (gas, electric, and kerosene) for cooking. Furthermore, it was observed in NFHS-3 that of those who used unclean fuel, i.e., coal, charcoal, crops residue, grass, dung cakes, etc., 91% used an open stove (chullah) not under a chimney.

### Anemia prevalence

Table 1 shows the prevalence of anemia by various predictor variables. Prevalence of anemia among children that lived in those households where there was no facility of toilet was found to be higher than those who had these facilities available. Type of house also made an important variable for anemia prevalence; high prevalence was seen among those children who lived in Kaccha or Semi Pucca houses. Children who stayed in households that used unclean fuel had higher anemic conditions than those in households that made use of clean fuel. 62.3% children reported to have symptoms of anemia were exposed to tobacco smoke in the house as against 60.9% children who did not experience tobacco smoking by anybody at home. Seasonality also had an effect on anemia; with a higher prevalence of anemia among children under-5 in summer and rainy season than in winter. Higher anemia levels could be seen among those children who lived in nuclear families than those who belonged to non-nuclear families.

**Table 1:** **Prevalence of anemia by selected characteristics among children under 5 years**.

Characteristics	Anemia in children under-5	
	%	*N*
**Environmental**	
Toilet facility	
Facility available	55.1	9628
No facility	63.9	19787
Type of house	
Kaccha	65.8	5329
Semi pucca	63.5	14396
Pucca	54.7	9614
Type of cooking fuel	
Clean fuel	51.6	4790
Unclean fuel	62.8	24697
House structure	
Nuclear	62.6	15201
Non-nuclear	59.2	15759
Smoking (anyone in the house)	
No	60.9	31823
Yes	62.3	512
Source of drinking water	
Improved	60.2	26496
Not improved	64.7	4399
Seasonality	
Winter	59.7	18815
Summer and rainy	62.9	13526
**Child**	
Sex of child	
Boy	60.2	16623
Girl	61.8	15718
Age of child (months)	
<6	67.3	3218
6–23	61.8	9080
24–59	59.1	17541
Birth order of child	
1	59.8	9609
2	59.4	8567
3	61.7	5296
4	63.4	8869
Stunting	
Normal	59.0	15189
Stunting	62.9	17160
Breastfeeding	
Never breastfed	61.5	1389
Less than 6 months	59.3	2023
6 months and above	59.0	13816
Still breastfeeding	62.9	14879
Taken drugs for parasitic infection	
No	61.3	26908
Yes	56.5	3079
**Socio-economic**	
Place of residence	
Urban	56.0	6956
Rural	62.4	24005
Caste	
Others	57.6	7965
OBC	62.8	6962
SC	72.4	3723
ST	59.2	12646
Religion	
Hindu	61.1	25583
Muslim	61.6	6349
Others	57.4	1372
Mother’s education	
No education	66.5	6945
Primary	63.6	2592
Secondary and above	55.7	7001
Mother’s working status		
Not working	60.6	22539
Working	61.9	9775
Wealth index	
Rich	53.5	9037
Middle	61.3	6484
Poor	65.7	16820
Region	
North	56.5	3980
Central	57.9	8933
East and north east	69.7	10956
West	56.3	3644
South	57.6	4827
**Media exposure of mother**	
Read newspaper or magazine	
No	63.6	24981
Yes	53.5	7330
Watch television	
No	65.2	15968
Yes	57.4	16371
Listen radio	
No	62.3	19967
Yes	59.0	12370

**Figure 3:**
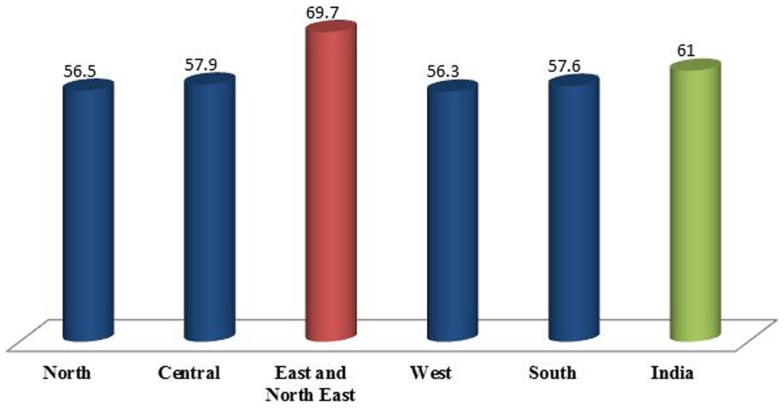
**Percentage of children under the age of 5 years having anemia by regions and India**.

Figure 3 shows the regional scenario of anemia prevalence among children under age of 5 years. At the all India level, the prevalence of the disease symptoms was 61%. However, some distinct regional variation was observed. Eastern along with the north eastern region had the maximum prevalence of anemia, i.e., 69.7%. Prevalence of anemia in North, Central, and West regions was around 56.5, 57.9, and 56.3%, respectively. The southern region had some of the best performing states in social indicators with the prevalence of anemia, around 57.6% compared to all other regions.

**Figure 4:**
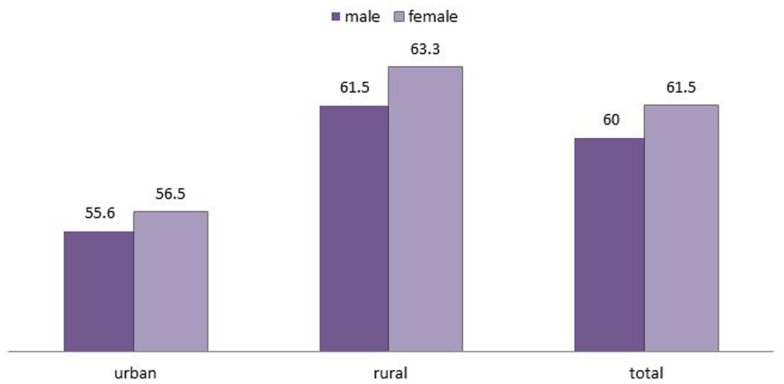
**Percentage of children under the age of 5 years who had anemia by place of residence according to sex**.

Figure 4 shows the percentage of children under the age of five years having anemia by place of residence according to sex (NFHS-3). The figure interprets that in both urban and rural regions of India, female children have higher percentage of anemia than male children which is also true for India as a whole.

**Figure 5:**
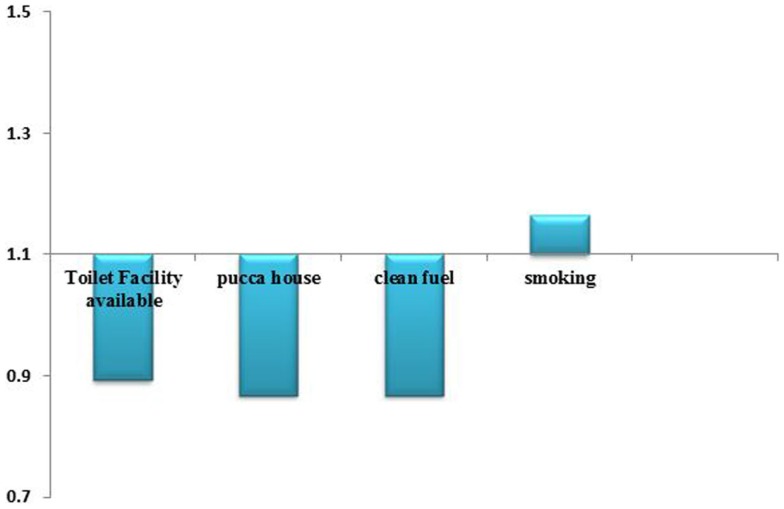
**Significant odds of suffering anemia by major household environment determinant**.

Figure 5 shows the significant odds of suffering anemia by major household environment determinants. It is inferred that with toilet facility available, pucca house and clean fuel, the odds of anemia in children under five years are decreasing while odds are increasing in case of smoking.

### Multivariate analysis

Table 2 shows the odds ratio of multivariate regression analysis based on children aged under 5 years of age. Among the environmental variables, in all the four regression models, it was seen that the odds of anemia among children living in households that had toilet facility had decreased as compared to the households, which had no such facility available. Similar results were found for the type of house and type of cooking fuel. Children living in pucca houses had a lower likelihood of anemia as compared to those living in Kaccha houses. Use of clean cooking fuel also reduced the chances of anemia among children significantly. Children that belonged to non-nuclear families had significantly lower chance of anemia compared to nuclear families. Improved or non-improved source of drinking water did not show any significant impact on the prevalence of anemia. After adjusting for socio-economic factors, smoking did not show any significant impact on anemia while adjusting with other factors smoking showed an increased impact on anemia among children. Seasonality did not show any noticeable impact on anemia.

**Table 2:** **Regression models showing odds of having anemia among children <5 years**.

Characteristics	Anemia in children under-5			
	Model 1	Model 2	Model 3	Model 4
**Environmental**	
Toilet facility	
No facility ^®^				
Facility available	0.825***	0.836***	0.880	0.893***
Type of house	
Kaccha ^®^				
Semi pucca	0.919***	0.922**	0.934	0.933
Pucca	0.813***	0.817***	0.877**	0.868**
Type of cooking fuel	
Unclean fuel ^®^				
Clean fuel	0.774***	0.794***	0.860***	0.868**
House structure	
Nuclear ^®^				
Non-nuclear	0.896***	0.897***	0.931**	0.935**
Source of drinking water	
Improved ^®^				
Not improved	0.985	0.996	0.923	0.945
Smoking (anyone in the house)	
No ^®^				
Yes	0.882	0.884	1.164***	1.164***
Seasonality	
Winter ^®^				
Summer and rainy	0.994	1.009	0.954	0.957
**Child**	
Sex of child	
Boy ^®^	–			
Girl		1.030	1.048	1.047
Age of child (months)	
Less than 6 months ^®^	–			
6–23 months		0.803***	0.804***	0.804***
24–59 months		0.734***	0.727***	0.725***
Birth order of child	
1 ^®^	–			
2		0.987	1.034	1.029
3		1.003	1.116	1.104**
4		1.061	1.033	1.023
Stunting	
Stunting ^®^	–			
Normal		0.898***	0.955	0.961
Breastfeeding					
Never breastfed ^®^	–			
<6 months		1.056	1.118	1.127
6 months and above		0.957	0.985	0.991
Still breastfeeding		0.978	0.924	0.929
Taken drugs for parasitic infection	
No ^®^	–			
Yes		0.893**	0.832***	0.838***
**Socio-economic**	
Place of residence	
Urban ^®^	–	–		
Rural			1.003	0.996
Caste
Others ^®^	–	–		
OBC			1.155***	1.151**
SC			1.331***	1.312***
ST			1.006	0.996
Religion	
Others ^®^	–	–		
Hindu			1.622***	1.602***
Muslim			1.552***	1.509***
Mother’s education	
No education ^®^	–	–		
Primary				0.918**	0.973
Secondary and above			0.776***	0.875
Mother’s working status	
Not working ^®^	–	–		
Working			0.942**	0.949
Wealth index	
Rich ^®^	–	–		
Middle			1.015	0.980
Poor			1.125**	1.057
Region	
North ^®^	–	–		
Central			0.926	0.953
East and north east			1.360***	1.383***
West			1.077	1.098**
South			1.091**	1.124**
**Media exposure of mother**	
Read newspaper or magazine	
No ^®^	–	–		
Yes				0.842***
Watch television	
No ^®^	–	–		
Yes				0.910**
Listen radio	
No ^®^	–	–		
Yes				0.939**
Constant	1.357	1.344	1.339	1.260
*R*^2^	0.015	0.019	0.034	0.036
*N*	536094	536094	536094	536094

*^®^ reference category*.

*p < 0.10.

**p < 0.05.

***p < 0.01.

Among the child variables, there were no significant odds of anemia by gender but with the increased age of children there was a significant decrease in the likeliness of anemia among children. With the increased birth order there was no change in the odds of anemia except in the fourth model where significant odds were found in the third birth order. There was not much difference in anemia prevalence among children with normal or stunted development. Similarly, the duration of breastfeeding also did not seem to affect the prevalence of anemia. Children who had taken drugs taken for parasitic infections, in the 6 months prior to the data collection, showed a decreased likelihood of anemia. Among the socio-economic variables, there was no impact of place of residence on anemia. There was a higher prevalence of anemia among OBC (Other Backward Classes) and SC (Schedule Caste) castes as compared to general castes. ST showed no significant change in odds of anemia. Hindu and Muslim children showed higher odds of being anemic than others. With the increased educational level of mother, there were reduced odds of anemia among children but this was not found in model 4. The working status of the mother had no significant impact on anemia. The poor showed higher chances of being anemic than the rich. Children in east, north east, and in southern regions showed significantly higher chances of anemia than those in other regions. Among the media exposure variables, there were significantly decreasing odds of anemia among those children whose mothers read newspaper, watched television, and listened to radio.

## Discussion

According to the World Health Organization (WHO); “*anaemia is a condition in which the number of red blood cells and in turn their oxygen-carrying capacity is insufficient to meet the bodies physiologic needs”* (5). From the present study, it was found that unclean fuel use, poor toilet facility, staying in non-concrete house, and smoking were important environmental variables determining anemia among children <5 years (6). According to National Rural Health Mission, India, poor environmental sanitation, unsafe drinking water, and inadequate personal hygiene conditions are some of the contributing factors toward anemia prevalence. The North and Eastern regions experienced significantly higher probability of having childhood anemia compared to all other regions of India (6). Report also states that more than half of the young children in 24 states of India have anemia, including 11 states where more than two third of children are anemic. Children who took drugs for parasitic infections showed lesser anemia prevalence in this study. National Rural Health Mission, India also confirms that parasitic infections like malaria and intestinal worms are one of the reasons for anemia among children in India (6). Anemia prevalence was found to be significantly higher in nuclear families. This could be because there is less number of members in the nuclear families to take care of children or because of the hectic schedule of family members. As a result, children do not get proper attention for food and nutrition. It was found smoking done by anybody in the house is significantly associated with child anemia. It is also evident from other studies that passive smoking in the household increases the risk of anemia in children (7).

## Conclusion

This study found that unclean fuel use, poor toilet facility, staying in non-concrete house, and passive smoking are the major determinants. Unclean fuel use mainly affects the health of pre-school children as they are more exposed to it. These children generally stay inside the households and are often carried by the mothers while cooking. Biofuel smoke contains high quantities of carbon monoxide, which binds with hemoglobin (transit oxygen to body tissues), forms carboxy hemoglobin, and reduces the oxygen-carrying capacity of hemoglobin, which leads to anemia. Clean drinking water, maintenance of sanitation, and hygiene practices contribute toward reducing anemia as they reduce parasitic infections among children. Therefore, there are multiple threats to anemia among children under the age of 5 years. Better resources are needed to educate the public and to increase awareness for improved hygiene, sanitation and housing facilities, health, and nutrition. Promoting and disseminating knowledge about the ill effects of passive smoking on the health of children can be an intellectual step toward it. Along with a wider program to manage nutritional deficiency anemia in children <5 years, there should be a holistic approach toward anemia control inculcating healthier household environmental conditions and improved socio economic factors.

## Conflict of Interest Statement

The authors declare that the research was conducted in the absence of any commercial or financial relationships that could be construed as a potential conflict of interest.
